# Durable response to afatinib in advanced lung adenocarcinoma harboring a novel NPTN-NRG1 fusion: a case report

**DOI:** 10.1186/s12957-023-03129-z

**Published:** 2023-08-16

**Authors:** Xin Nie, Ping Zhang, Zhixin Bie, Chenhui Song, Min Zhang, Di Ma, Di Cui, Gang Cheng, Hui Li, Yan Lei, Xiaoxing Su, Wendy Wu, Lin Li

**Affiliations:** 1 Department of Medical Oncology, Beijing Hospital, National Center of Gerontology, Institute of Geriatric Medicine, Chinese Academy of Medical Sciences, No. 1 DaHua Road, Beijing, 100730 China; 2 Department of Minimally Invasive Tumor Therapies Center, Beijing Hospital, National Center of Gerontology, Institute of Geriatric Medicine, Chinese Academy of Medical Sciences, Beijing, China; 3 Berry Oncology Corporation, No. 4 Science Park Road, Beijing, 102206 China; 4 Department of Radiology, Beijing Hospital, National Center of Gerontology, Institute of Geriatric Medicine, Chinese Academy of Medical Sciences, Beijing, China; 5 Department of Pathology, Beijing Hospital, National Center of Gerontology, Institute of Geriatric Medicine, Chinese Academy of Medical Sciences, Beijing, China

**Keywords:** NRG1 fusion, Afatinib, Lung cancer, Next-generation sequencing (NGS)

## Abstract

**Background:**

NRG1 fusions are rare oncogenic drivers in solid tumors, and the incidence of NRG1 fusions in non-small cell lung cancer (NSCLC) was 0.26%. It is essential to explore potential therapeutic strategies and efficacy predictors for NRG1 fusion-positive cancers.

**Case presentation:**

We report an advanced lung adenocarcinoma patient harboring a novel NPTN-NRG1 fusion identified by RNA-based next-generation sequencing (NGS), which was not detected by DNA-based NGS at initial diagnosis. Transcriptomics data of the tissue biopsy showed NRG1α isoform accounted for 30% of total NRG1 reads, and NRG1β isoform was undetectable. The patient received afatinib as fourth-line treatment and received a progression-free survival (PFS) of 14 months.

**Conclusions:**

This report supports afatinib can provide potential benefit for NRG1 fusion patients, and RNA-based NGS is an accurate and cost-effective strategy for fusion detection and isoform identification.

**Supplementary Information:**

The online version contains supplementary material available at 10.1186/s12957-023-03129-z.

## Introduction

Neuregulin 1 (NRG1) gene fusions are rare oncogenic drivers across multiple solid tumors, and the incidence of NRG1 fusions in non-small cell lung cancer (NSCLC) was 0.26% [1]. In NRG1 fusion-positive lung cancers, majority of gene partners were identified in upstream regions (84%), and the most common upstream partners were CD74 (41%) and SLC3A2 (20%) [2]. NRG1 fusions were mostly detected by RNA-based testing [2]. Accurate molecular diagnosis provides insight into the mechanism of malignant alteration and contributes to the decision of treatment strategy.

NRG1 contains an epidermal growth factor (EGF)-like domain and mainly binds to ErbB3 receptors which are activated by heterodimerization with ErbB2 [3]. Limiting the activity of ERBB2-ERBB3 heterodimer may be the most effective strategy for NRG1 fusion-positive cancers. Due to the low incidence of NRG1 fusions and various fusion partners, additional research is needed to further understand the biology and therapeutic strategies of NRG1 fusion-positive cancers. Herein, we report a case of advanced lung adenocarcinoma harboring a novel NPTN-NRG1 fusion, which achieved durable response to afatinib.

### Case report

A 54-year-old Chinese female non-smoker admitted to the hospital in June 2020 for progressive dyspnea. The patient had no family history of cancer. Chest computed tomography (CT) identified a right upper lobe mass with interlobular pleural metastasis and mediastinal lymphadenopathy. No distant metastasis was revealed by bone scan, head, and abdominal CT. CT-guided trans-thoracic core-needle biopsy of the right lung mass demonstrated poorly differentiated adenocarcinoma. Immunohistochemistry (IHC) staining revealed the pulmonary tumor was positive for TTF-1 and negative for VENTANA ALK (D5F3) and P40. Tumor proportion score (TPS) for programmed death ligand 1 (PD-L1) was 2%. A DNA-based next-generation sequencing (NGS) assay showed negative for driver gene alterations, microsatellite stability (MSS), and tumor mutation burden (TMB) was 6.7 Muts/Mb in the pulmonary tumor. The patient was diagnosed with poorly differentiated adenocarcinoma of right upper lobe, stage IVA (cT1cN2M1a).

The patient received pemetrexed plus carboplatin combined with PD-1 inhibitor toripalimab and switched to pemetrexed plus toripalimab as maintenance therapy between July 2020 and February 2021, which achieved partial response (PR). In March 2021, CT scans showed disease progression in both primary tumor and pleural metastases. She received paclitaxel and toripalimab as second-line therapy. However, CT scans showed pleural metastases increased after two cycles of treatment. She was transited to toripalimab and anlotinib, as third-line treatment since May 2021, and the best response was stable disease (SD). Both primary tumor and pleural metastases progressed in December 2021.

**Fig. 1:**
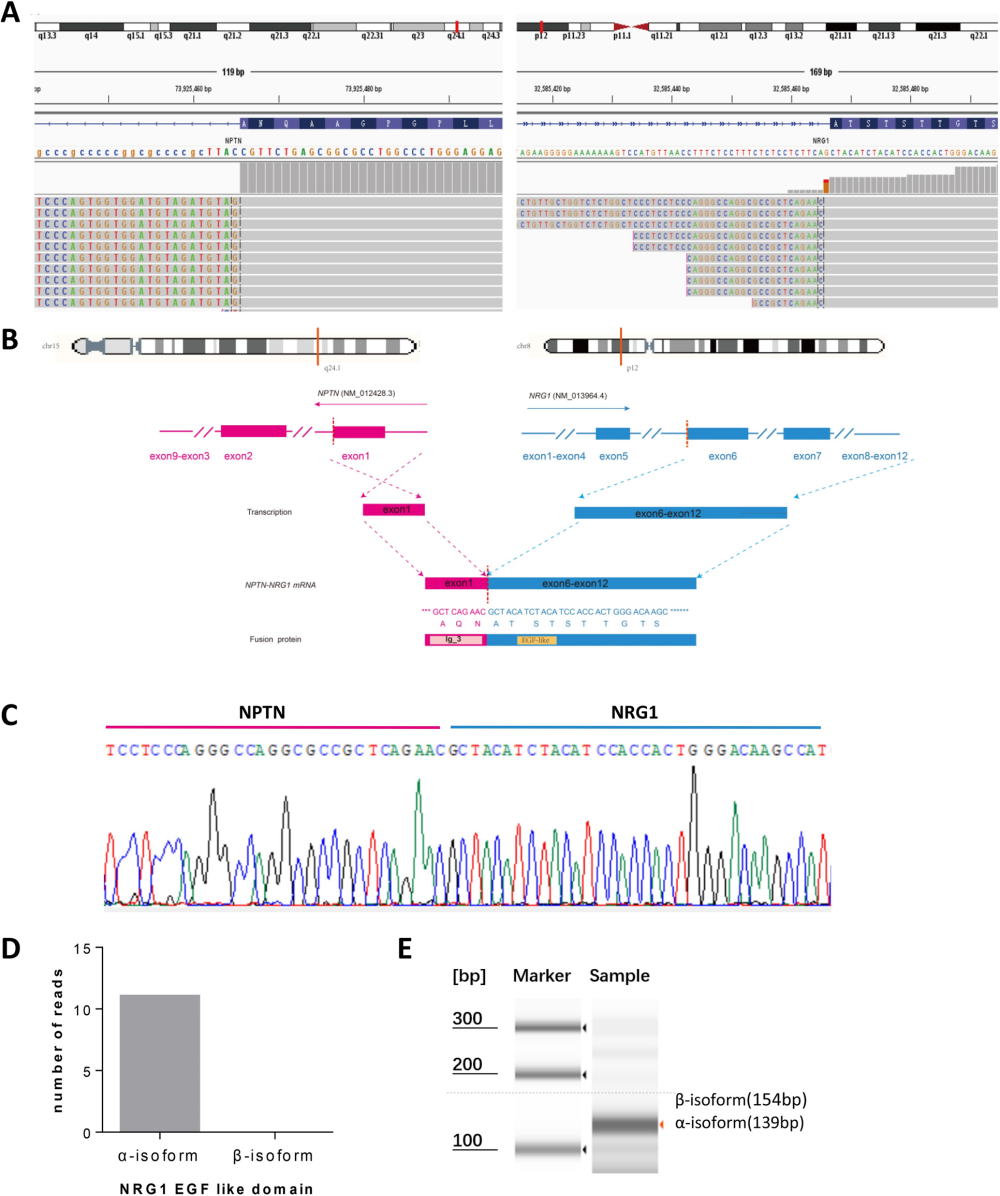
Identification and verification of a novel NPTN-NRG1 fusion. **A** RNA sequencing reads indicating NRG1 fusion regions were visualized by the Integrative Genomic Viewer (IGV) software. **B** Schematic of genomic rearrangement involving the fusion breakpoints in mRNA and protein level. **C** Sanger sequencing confirmed the NPTN-NRG1 fusion transcript and showed the breakpoint sequence. **D** The number of NRG1 EGF-like domain α or β isoform-specific read in RNA-based NGS data. **E** Reverse transcriptase-polymerase chain reaction (RT-PCR) confirmed the *α* or *β* transcript variants of NRG1

**Fig. 2:**
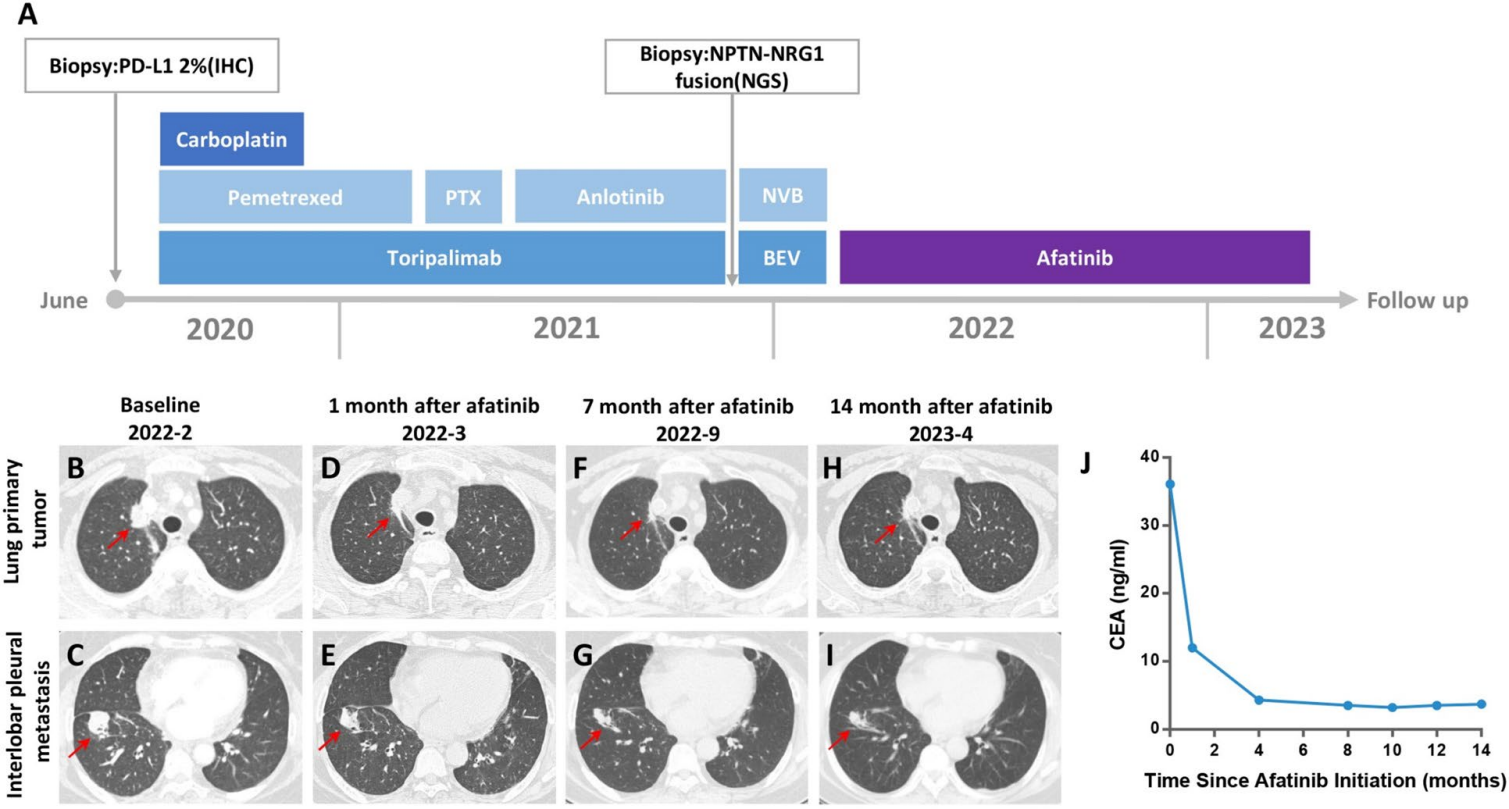
Treatment timeline and clinical response of the NRG1 fusion-positive patient with afatinib. **A** Timeline of the patient’s clinical treatment course. **B**–**I** Computed tomography (CT) scan of the chest in different afatinib treatment stages, CT images of lung primary tumor (**B**), and interlobar pleural metastasis (**C**) at baseline before afatinib therapy. Primary tumor and interlobar pleural metastases shrinked after 1 month (**D**,** E**), 7 months (**F**,** G**), and 14 months (**H**,** I**) of afatinib treatment. **J** Dynamic changes of carcinoembryonic antigen (CEA) during afatinib therapy. BEV, Bevacizumab; NVB, Navelbine; PTX, Paclitaxe

In December 2021, the patient received biopsy of pleural lesion, followed by a targeted DNA and RNA based NGS testing which encompasses 654 cancer-related genes (Berry Oncology). Targeted NGS assay showed a NPTN-NRG1 fusion and CCND1 gene copy number variations without other driver mutations (Fig. 1A and B). The breakpoints and fusion sequence were confirmed using reverse transcriptase-polymerase chain reaction (RT-PCR), followed by Sanger sequencing (Fig. 1C). NGS detection showed MSS and TMB was 6.27 Muts/Mb. PD-L1 expression was negative (TPS < 1%). In order to reduce the risk of adverse events from PD-1 inhibitor followed by epidermal growth factor receptor tyrosine kinase inhibitors (EGFR-TKIs), the patient completed two cycles of navelbine plus bevacizumab. However, pleural metastases still increased. Afatinib 40 mg/day was initiated since February 3, 2022. In March 2022, PR was achieved and the chest CT scan showed significant decrease of both primary lesion and pleural metastases (Fig. 2 B–I). Carcinoembryonic antigen (CEA) decreased from 36.2 ng/mL before afatinib to 12 ng/mL in March 2022 and decreased to normal level since June 2022 (Fig. 2J). The patient was well tolerated with afatinib and only experienced grade 1 diarrhea and rash. Delayed immune-related adverse events and recurrence were not observed until now (April 6, 2023), reflecting a progression-free survival (PFS) of 14 months.

## Discussion

In this case study, we reported a novel NPTN-NRG1 fusion in lung adenocarcinoma. Neuroplastin (NPTN) is a glycoprotein that belongs to the immunoglobulin (Ig) superfamily of cell adhesion molecules. There were few studies investigating the function of NPTN in cancer. An in vivo experimental study substantiated over-expression of NPTN can cause significant increase in tumor growth and angiogenesis [4]. NPTN has not been reported as a fusion partner of NRG1 or any common fusion gene. To our knowledge, this is the first report of NPTN-NRG1 fusion in lung adenocarcinoma.

In our case, NPTN as the upstream fusion partner retained the Ig-like 3 domain, and NRG1 retained the EGF-like domain. Several studies showed ERBB2 and ERBB3 inhibitors and monoclonal antibodies were effective in solid tumors harboring NRG1 fusion and the most frequently reported one was afatinib, an irreversible pan-ErbB family inhibitor. In a real-world multicenter study, afatinib was used to treat 20 patients with NRG1 fusion-positive lung cancers, achieving an objective response rate of 25% and a median PFS of 2.8 months [2]. It indicated that some patients with NRG1 fusions could benefit from ERBB-targeted therapy and some not. Hence, identifying predictive factors for ERBB targeted therapy in NRG1 fusion cancers may help to guide clinical decision.

Changes in spliced transcripts are a hallmark of cancer and provide a novel perspective on cancer prognosis. NRG1 have two major splice alternatives in their EGFR-like domains, α and β isoform. NRG1β has higher affinity to ERBB than NRG1α [5]. The expression of NRG1α and NRG1β presented no significant difference in normal sample, but varied in tumor samples [6]. In our case, the main type of NRG1 isoform was NRG1α accounting for 30% of total NRG1 reads and NRG1β was undetectable, which was detected by RNA-based NGS test (Fig. 1D) and confirmed by RT-PCR (Fig. 1E). This patient received a durable response to afatinib, which may be associated with NRG1 isoforms. There were few studies investigating the relationship between NRG1 isoforms and prognosis. A previous study showed that high level of NRG1α was associated with favorable survival in breast cancers [7]. A case showed a patient with stage IB NRG1-positive lung cancer expressing significant higher level of NRG1β had an overall survival (OS) of 39 months [8], which was much shorter than the median OS of patients with stage I NRG1-positive lung cancers [2]. Another study showed the PFS of EGFR-TKIs for two NRG1 fusion-positive metastatic pancreatic adenocarcinoma with higher β isoform level was only 3 and 5 months, respectively [9]. Thus, high level of the lower affinity EGF-like domain, α isoform, might be biologically less aggressive or more responsive to treatment, which needs to be evaluated further. Besides, RNA-based NGS can detect expressed fusion transcripts directly and avoids the difficulties of sequencing larger intronic regions, which also offers a convenient detection approach to identify transcript isoforms.

In conclusion, this report identified a novel NPTN-NRG1 fusion in a heavily treated patient with advanced lung adenocarcinoma with durable response to afatinib. It highlights that DNA and RNA-based NGS panel is a cost-effective solution to fusion gene detection.

### Supplementary Information

**Additional file 1.** The sequence of primers for RT-PCR.

## Data Availability

All data and materials generated or analyzed during this study are included in this article. Further information can be obtained from the corresponding author on reasonable request.
