# Supplementary material for: Durable response to afatinib in advanced lung adenocarcinoma harboring a novel NPTN-NRG1 fusion: a case report
Source: World J Surg Oncol. 2023 Aug 16;21:246. doi: 10.1186/s12957-023-03129-z (PMC10428614; doi:10.1186/s12957-023-03129-z)
Supplement: Supplementary file 1 — Additional file 1. The sequence of primers for RT-PCR. [file 12957_2023_3129_MOESM1_ESM.docx]

**Supplementary Material**

Supplementary Table 1. Sequence of primers for reverse transcriptase-polymerase chain reaction (RT-PCR)

| **gene** | **Forward** | **Reverse** |
| --- | --- | --- |
| NRG1 | GGAGTGCTTCATGGTGAAAGACC | CTGGTACAGCTCCTCCGCC |
